# Self-Monitoring Risk Factors for Diabetic Foot Ulceration With the Feetchecker App: Mixed Methods Study

**DOI:** 10.2196/80769

**Published:** 2026-05-27

**Authors:** Roy van den Heuvel, Mark Arts, Deborah Pelders, Rens Brankaert

**Affiliations:** 1 Department of Industrial Design, Eindhoven University of Technology, PO Box 513, Eindhoven, 5600MB, The Netherlands, +31 885072576; 2 Allied Health Professions, Fontys University of Applied Sciences, Eindhoven, The Netherlands; 3 Tranzo, Tilburg University, Tilburg, The Netherlands

**Keywords:** diabetes mellitus, diabetic foot ulcer, prevention, mHealth, mobile health, eHealth, Feetchecker

## Abstract

**Background:**

A prevalent and serious complication of diabetes mellitus is the development of diabetic foot ulcer (DFU). There is a need for effective solutions that help prevent DFU to support our increasingly stressed health care systems. The use of mobile health (mHealth) tools has been shown to improve awareness and effective self-care management skills in people at risk of developing diabetic foot ulceration.

**Objective:**

In this study, we aimed to investigate the perceived usefulness, engagement, and overall user experience of the Feetchecker app, a self-monitoring mHealth app for people at risk of DFU.

**Methods:**

A total of 24 patients (mean age 71, SD 8.6 years) with type 2 diabetes mellitus at risk of developing diabetic foot ulceration completed a 3-month evaluation period (70 recruited, 36 included, 12 dropped out) of a self-monitoring mobile app called Feetchecker app. A mixed methods approach was used to combine insights from app data with qualitative data from a pre- and postsurvey as well as interviews with patients and involved podiatrists. Data were analyzed using descriptive statistics and thematic analysis. We evaluated overall use of the app, patient engagement, and user experiences.

**Results:**

Patients who fully completed the study conducted 393 feetchecks. In total, 7 patients sent in 9 pictures; all 7 were called for follow-up by a podiatrist. Overall, patients had a positive experience with the app and perceived the Feetchecker app as a valuable tool to monitor their feet for potential risk factors of DFU. Ease of use in performing a feetcheck and sending the podiatrist a picture was described as an important feature. Three main types of engagement with the Feetchecker app emerged: continuous, frequent, and no to little engagement. These patterns highlight enablers for self-monitoring such as ease-of-use, easy access to a podiatrist, and social support, as well as barriers such as digital skills and sustained engagement. Podiatrists highlighted the benefits of having patients report potential issues quicker and the ability to monitor their patients remotely. Challenges remain in integrating the promotion of the Feetchecker app into their consultations.

**Conclusions:**

The Feetchecker app supported patients in self-monitoring risk factors associated with DFU through routine checks and quick contact with a health care professional in case of a potential issue. Overall, patients described a positive user experience and considered the app helpful. While mHealth tools are not for everyone, user engagement for many patients was high and shows that such apps can offer support for people able to use them. Future research should focus on improving usability and engagement with the app as well as extend the way patients can communicate with health care professionals beyond a picture.

## Introduction

Diabetes mellitus ranks among the most prevalent chronic conditions globally, with projections indicating a significant rise in the years ahead [1]. A common and serious complication associated with diabetes is diabetic foot ulcers (DFUs). The development of DFU is closely linked to peripheral neuropathy [2], foot deformities, and varying degrees of ischemia [3]. Furthermore, diabetes-related foot ulcers markedly heighten the risk of infection, leading to increased rates of lower extremity amputations, diminished quality of life, higher mortality, and health care costs [4]. Regular screening, education, and self-management are essential strategies recommended to prevent the onset of diabetic foot complications [5]. In the Netherlands, specialized podiatrists primarily provide foot care for patients with diabetes. However, with the rapid increase in the diabetic population, the availability of podiatrists is anticipated to fall short of meeting the growing care needs. Consequently, there is an urgent demand for preventive measures such as self-monitoring and remote care services.

Self-monitoring for diabetes can contribute to effective and cost-efficient care by reducing glycated hemoglobin levels and diabetes-related complications [6-8]. This approach includes raising awareness of potential complications like DFU [9], promoting self-management strategies [10,11], and using digital platforms to provide insights into self-care practices [12]. Research has shown that the use of mobile health (mHealth; and telemedicine) apps can improve knowledge and self-care management skills in people at risk of DFU [13-16]. In a review of telemedicine tools for this target group, Søndergaard et al [17] describe how such solutions gave patients an experience of having more control over their lives and a feeling of empowerment. In a qualitative systematic review, Foong et al [18] describe facilitators and barriers toward using digital technologies aimed at managing DFU. Facilitators such as information sharing and wound image taking are highlighted as enablers toward prevention and personalized care. Important barriers such as a lack of face-to-face care, usability for older users, and a potential lack of patient engagement in asynchronous consultations were identified as avenues for improvement.

Building on these facilitators and barriers, recent research by Ploderer et al [19] has highlighted the effectiveness of a smartphone app called MyFootCare, designed to aid in the self-monitoring of the DFU healing process through tracking the size of an existing wound. While the picture-taking and wound-tracking features were promising, data sharing between patients and care professionals and prevention-focused functionalities were lacking. Another study that focused on the clinical monitoring of DFU created the Foot Selfie system [20]. While this work showcased benefits such as asynchronous communication between health care professionals and patients through the daily picture upload, the study also focused primarily on clinical use. Both Ploderer et al [19] and Swerdlow et al [20] showed that the ability to track improvements and see data reflecting a reduction in DFU incidence can serve as a strong motivational factor for patients to engage more actively in their self-care routines.

While managing existing DFUs and wound healing is crucial, pre-emptive measures to prevent their occurrence in the first place are critical. A study by Kilic and Karadağ [21] focused on this preventative effort through their “Mobile Diabetic Foot Personal Care System” app. Their results suggest that their self-monitoring app improved self-efficacy and increased knowledge about the diabetic foot and its management. The study instructed patients to use the app daily, and every time they measured their blood glucose levels, they were asked to also send in foot observations. While this increased the number of completed checks, it is unclear how it integrates into patients’ own everyday routines. Understanding how the needs of people with diabetes differ between individuals and change over time is important to fit self-management interventions to people’s routines [22]. Research is needed to help people prevent ulceration through self-monitoring at home, particularly regarding the usability of these mHealth tools and how people integrate DFU self-monitoring into their everyday routines over time.

To address these challenges, the “Feetchecker app” was developed by a large podiatry organization in the Netherlands to support patients in everyday monitoring of potential foot-related issues. These could be, for example, (small) lesions or discolorations, all aimed toward the prevention of DFU. The app was created during the early stages of the COVID-19 pandemic in 2020. The podiatry organization wanted to maintain their culture of care, which they describe as putting patients “at the center of their care,” meaning they would consider their wishes and needs, and maintain personal contact with podiatrists. The app works as follows: it sends notifications to patients with diabetes at risk for foot ulceration to check their feet, guided by questions and instruction videos. Patients take a picture of their feet if the answers to the questions indicate that something is wrong. The picture is then inspected remotely by a podiatrist specialized in diabetes care for the presence of (minor) lesions, which indicate a strongly increased risk for ulcer recurrence [23]. If required, the patient will be contacted by their own podiatrist for follow-up. An initial informal evaluation of the first version of the Feetchecker app showed that patients appreciated the ability to share pictures in an easy way and direct contact with the podiatrist if something was wrong. However, app engagement was low, and due to the quick implementation, it was missing essential features for sustainable use. Through a robust co-design process [24], the app was redesigned from the ground up to better align with the needs of patients and the reality of podiatry practice, which resulted in a new design of the app and a picture backend system for the podiatrists.

The purpose of this mixed methods study is to evaluate the perceived usefulness, patient engagement, and overall user experience of the Feetchecker app system with patients and podiatrists and provide implications for foot health monitoring apps.

## Methods

### Study Design

This study was structured as a mixed methods prospective cohort study over a 3-month period. In this study, we integrate quantitative app use data with rich qualitative insights from participants and podiatrists. The study focused primarily on the used qualitative methods, which were based on open-ended responses from surveys and narratives from interviews. The study focused on usefulness, user experience, and engagement with the Feetchecker app. In this study, we conceptualized engagement as “effective engagement” [25,26], in which we describe the total number of times the app was used, the duration (for how long the app was used), frequency (eg, patterns of use), and depth (eg, use of the knowledge clips) [27]. Additionally, we consider user experience as satisfaction, acceptability, credibility, perceived impact, and usability [28].

### Intervention

**Figure 1.**
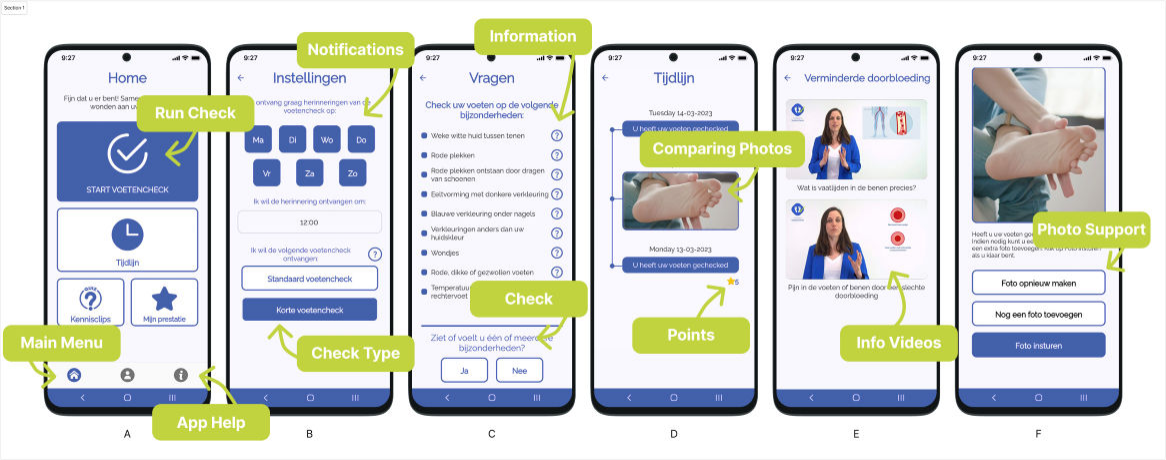
Six annotated screenshots of the Feetchecker app, screens (A-F). Screen (A) shows the home screen. Screen (B) shows the settings screen with the notification options. Screen (C) shows the short Feetchecker checklist. Screen (D) shows the timeline of previous checks. Screen (E) shows informative videos and quizzes. Screen (F) shows the picture submission screen. More detailed descriptions of the features are in Multimedia Appendix 2.

The Feetchecker app (Figure 1) was designed through an iterative, user-centered co-design process, including multistakeholder cocreation sessions with patients, therapists, and a variety of other health care professionals. This process is described in detail in Pelders et al [24]. This led to the final version of the Feetchecker app, which was developed into a fully functioning iOS and Android app [29]. To use the app, users need to use a specific patient number, which they obtain from the clinic that uses the Feetchecker app. In addition, patients need to enter their date of birth; this acts as an additional identity check. Data and pictures from the Feetchecker app are linked to the right patient record in the electronic health record (EHR) system so the podiatrist can identify the patient for follow-up when required. The core features include the 9-question feetcheck, which asks questions such as whether patients see a red spot, cut, or sense temperature differences (Multimedia Appendix 1), the picture-taking module, a timeline with the history of completed checks and pictures, curated knowledge clips created with podiatrists specialized in diabetes, setting reminders, and frequently asked questions. A more detailed description of the intervention and its features can be found in Multimedia Appendix 2. Podiatrists let patients decide their own check routines. Most podiatrists instruct patients to “use it when you experience a problem.”

The Feetchecker app does not fall under the European Medical Device Regulation [30], as the app has no intended medical purpose and does not perform any medical action (eg, advice and diagnostics) on the collected data different from storage, archival, and communication.

### Recruitment

#### Eligibility

Eligible participants were patients of Rondom Podotherapeuten with diabetes mellitus, without active DFU. Eligibility required owning a smartphone to access the app, a sufficient understanding of the Dutch language, and the ability to independently provide informed consent. During the entire study period, patients continued to receive standard care from their clinic.

#### Recruitment

We recruited through Dutch clinics from Rondom Podotherapeuten. Podiatrists recruited patients who either came in for a regular consultation or were new patients. Patients were recruited for a period of 3 months. Podiatrists checked eligibility criteria and explained the app and the aim of the study. If patients accepted participation and consented to being contacted by the researchers (allowing their contact information to be shared), podiatrists registered this in their EHR.

#### Inclusion

Contact details were then relayed to the researchers, who contacted the patient either via phone or mail and asked permission to send the information letter (package) and digital consent form. Once patients signed the informed consent form, they were included in the study. Throughout the study, the patients received care as usual; the Feetchecker app functions as a complementary instrument, not as a way of replacing traditional care.

#### Sample Size

This study aimed to recruit 70‐80 patients to gain a good, qualitative understanding of the patient experience in using the Feetchecker app. We anticipated a dropout rate of around 50%, based on earlier experiences from the pilot [24].

### Ethical Considerations

Ethics approval of the study was granted by the institutional ethical committee of the Fontys University of Applied Science and approved by the board of the clinic. All participants (patients and podiatrists) were provided with an information letter describing study details. Informed consent was obtained from all participants. This included consent to publish anonymized research data such as interview results and Feetchecker pictures. All study data were pseudonymized using a patient code to ensure confidentiality. Research data required for the study were password-protected and stored at the research institute’s secure storage facility, following General Data Protection Regulation standards. Study data were deidentified after the study was finished. Only the research team had access to the decryption key. The research team had no access to the EHR system of the clinic. Participants did not receive any form of compensation for participation. Throughout the study period, a dedicated phone number and email address were available for patients to contact the researchers for technical support or with any queries related to the study or the Feetchecker app.

### Data Collection

The mixed methods data collection strategy consisted of demographic baseline characteristics of the patients, an intake and poststudy survey and semistructured interviews with patients and podiatrists, and finally, quantitative data from the app server data logs. All qualitative data were first analyzed in Dutch and subsequently translated into English for publication. Analyses of the qualitative data were done by 2 of the authors (RH and DP).

#### Demographics Collection

The primary demographic data of patients, including age, Care Profile, and sex, were collected by the clinic from EHRs, encrypted, and sent to us. Care Profiles are a Dutch classification of care needs. Patients with a higher Care Profile indexation often have had previous problems with their feet and thus might understand the use of a preventative app better or might have already received instructions for checking their feet more often. For more details on Dutch podiatric practice and care profiles, see Multimedia Appendix 3. All communication (email, survey, etc) was written in Dutch and was checked by a senior researcher (RB) with expertise in conducting research with patients in the age range of our target group.

#### Feetchecker App Data

Quantitative data were collected from the Feetchecker app database, which contained entries such as Feetchecker question responses, submitted pictures, and clinic responses. A podiatrist specialized in diabetes care described the clinical reasoning process behind these pictures, which can be found in Multimedia Appendix 4.

#### Surveys

##### Intake Survey

Upon enrollment, patients received an email that contained a link to the intake form in Microsoft Forms (Multimedia Appendix 5) that included the information letter and consent form. After signing, patients were asked to complete a survey, which focused on their current foot health routines, if they had used the Feetchecker app before, and what their initial experiences with the app were. If the patient preferred a physical information booklet and survey, they could indicate that to the podiatrist, who had physical copies available.

##### Poststudy Survey

At the end of the 3-month study period, patients were asked to complete a postsurvey (Multimedia Appendix 5). This survey was designed to evaluate the app in terms of engagement and user experience and consisted of 23 questions (a Likert scale and the possibility to elaborate qualitatively).

### Interviews

#### Poststudy Interviews With Patients

At the end of the postsurvey, patients had the option to consent to be contacted for a phone-based interview. Semistructured interviews (maximum 30 minutes) were conducted based on an interview guide, which can be found in Multimedia Appendix 6. These interviews aimed to deepen our understanding of their experiences by capturing rich, qualitative data about how they used the app. The interviews were audio-recorded and transcribed verbatim.

#### Semistructured Interviews With Podiatrists

In addition to interviewing patients, semistructured interviews (30‐45 minutes) were conducted with the podiatrists involved. These discussions were intended to gain insights into how podiatrists perceived their patients’ use of the app, their experiences in assessing the images submitted through the app, and the overall integration of the Feetchecker app during podiatry visits. The interviews were audio-recorded and transcribed verbatim.

### Data Analysis

#### Thematic Analysis

The study used a thematic analysis approach [31] to explore and interpret the qualitative data collected. The analysis was conducted by a multidisciplinary team comprising design researchers, clinical researchers, and podiatrists, all of whom are proficient in digital literacy and familiar with eHealth technologies. This expertise, however, introduces potential biases, particularly in terms of technological predispositions. Interviews were transcribed and coded, and themes were created through extensive discussions with researchers and podiatrists. The patient group primarily consisted of Dutch-speaking patients, as the app was only offered in Dutch.

#### Quantitative Data Analysis

All quantitative data from the surveys, demographics, and app data were analyzed using Microsoft Excel. We use descriptive statistics to share general results.

## Results

We first describe the overall use of the app through app data logs, submitted pictures, and follow-up calls. Then, we describe and visualize user engagement over time, which we further enrich with app experiences through qualitative data analysis.

### Participants

In total, we recruited 70 patients through the podiatry clinics, of which we could include 36 patients, who then signed the consent form and started the study. In total, 24 patients (male: n=20, female: n=3, unknown: n=1) with an average age of 71 (SD 8.6) years completed the exit survey and concluded the study. Detailed demographics per participant can be found in Multimedia Appendix 7. All patients successfully installed the Feetchecker app on their personal smartphones. Of all patients who completed the study, 10 consented to a follow-up interview, of which, we could reach 5 for an interview over the phone, which lasted ~20‐40 minutes. All patients had type 2 diabetes mellitus. Most patients had a high risk of developing foot complications, indicated by the Care Profile, which is a classification of care needs further explained in Multimedia Appendix 3. Of the 24 patients, we had 9 very high, 12 high, 1 medium, and 2 unknown risk profiles. The average study period for the patients was 111 (SD 19.9) days. Not every patient filled in the exit survey immediately after 3 months, causing their study period to be longer than 3 months, hence, the variability in study duration.

Reasons for dropping out were mostly due to not responding to invitations and reminders to complete either the start or exit survey and reminders for starting the study after recruitment. One patient indicated that they no longer wanted to participate due to a lack of digital literacy.

Additionally, we interviewed 5 experienced (8‐20 years) podiatrists, familiar with treating patients with diabetes, who helped recruit patients for the study and who had experience with the Feetchecker app.

### Quantitative Results

#### Feetchecker App Data

Patients who completed the study fully (including exit survey) conducted 393 feetchecks. In total, 7 patients sent in 9 pictures, all 7 were called for follow-up.

#### User Engagement Data

**Figure 2.**
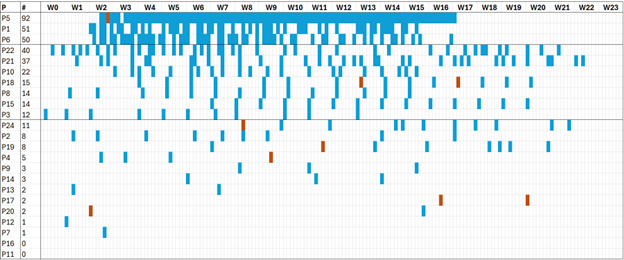
A visual overview of submitted feetchecks and pictures per patient over the entire duration of the study. The chart is organized to show the patient with the highest number of checks at the top. The blue marks are feetchecks executed by patients (**P**) over the study period ( week [W] 0-23). The chart is sorted by the number of checks (#). A check that resulted in taking and submitting a picture is displayed as a red mark.

Figure 2 shows the Feetchecker logs per patient throughout the evaluation period.

**Figure 3.**
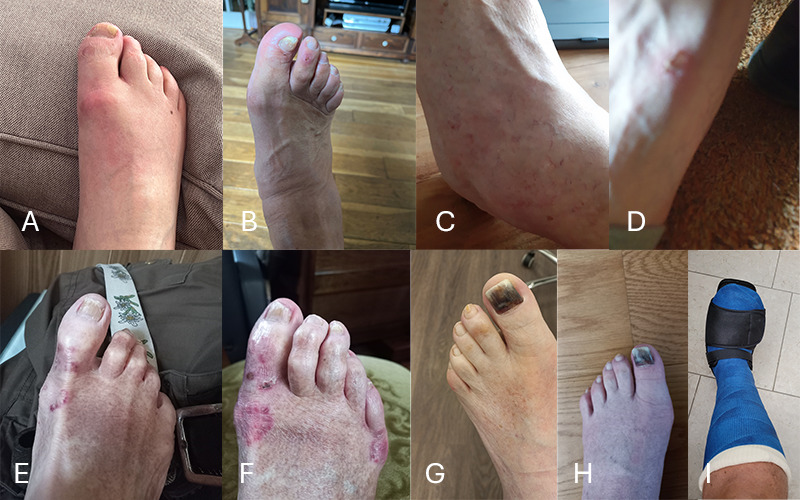
A collage of the 9 submitted Feetchecker pictures (**A-I**). Every picture shows a potential issue at the foot of the patient. Figure 3G and H are pictures of the foot from the same person. Detailed descriptions of all pictures by a podiatrist specialized in diabetes care are available in Multimedia Appendix 4.

The data presented in Figure 3 show that 3 patients have consistent, high use of the app, conducting a feetcheck almost every 1 or 2 days. Then, 7 patients consistently use the app once or twice a week for the study period. This is in line with how podiatrists often suggest the app to be used (once per week or how patients see fit). Then, a group of 11 patients who are less consistent in use, where some use the app only a few times. The last 2 patients did not use the app at all.

#### Feetchecker Picture Data

Figure 3 shows the pictures that the patients took of their feet during the study. From the set of 9 pictures, we observed that patients took pictures from various angles, highlighting specific parts of the foot, such as a plantar view of the forefoot, the dorsum, or the tip of the toes (Figure 3). The pictures in the data show differences in quality (eg, light, focus, and composition). For example, Figure 3C is dark, and Figure 3D is unfocused. Assessing these images often costs podiatrists more time or requires them to contact the patient.

In the clinical descriptions of the podiatrist specialized in diabetes care, which can be found in Multimedia Appendix 4, we identify various reasons to contact the patient. First, in Figure 3A, B, E, and F, the podiatrist identifies a potential problem such as indicators for pressure sores or bruises. The podiatrist needs to follow up with the patient to identify the issue and prevent worsening. Second, in Figure 3C, G, and H, the podiatrist recognizes the need to provide information. The picture does not show a problem, but patients might need to be comforted and provided with information about the cause of, for example, the discolorations. Third, for Figure 3D and I, the podiatrist is simply curious about what is in the picture (eg, due to a blurry image) and calls to understand what the cause is and can then provide advice.

### Qualitative Results

#### Overview

Four domains emerged from our analysis: (1) patient experience, (2) podiatrist experience, (3) impact of education and instruction, and (4) barriers and limitations in app use. Within these domains, we identified several themes that we illustrate with quotes from the interviews with patients and podiatrists as well as the surveys.

1. Patient experience
  - Patient motivations to check their feet using the checklist
  - The experience of taking pictures with the app
2. Podiatrist experience
  - Podiatrist perspective on the value of the app for their patients
  - Inspecting the Feetchecker images
  - Podiatrist workflow with the Feetchecker app
3. App education and instruction
  - Instructions on how to use the Feetchecker app
  - Patient education through the Feetchecker app
4. Barriers and limitations in app use
  - Barriers toward adopting digital care apps
  - Accessibility and digital skills

The results from the evaluation show that 19 of 24 (80%) patients found the Feetchecker app helpful in inspecting their feet, as one patient highlights: “... I can’t really do it without the app, it’s a real reminder for the health of my feet” (Patient 10).

#### Theme 1: Patient Experience

##### Patient Motivations to Check Their Feet Using the Checklist

Most patients primarily used the checklist and sometimes the picture-taking functionality. While patients often check their feet without the app, the 9-point checklist helped them look for potential issues they often did not check for: “You know, you almost never look for [those] things, so that’s the nice thing, you get more points of attention than I would look for by myself” (Patient 1).

Over half of the patients (15/24, 80%) frequently (daily or 1‐3 per week) used the app and kept up that engagement over time, as one patient says: “Gives me a sense of regularity to check my feet” (Patient 8). Although few patients mentioned that they actively set the reminders to times they preferred (beyond the default), they recognized that without the reminders, they would do it less: “Without the app, I would do it way less” (Patient 15). While the app (reminders) helps patients to regularly check their feet, 9 (37%) users use the app rarely (less than once a week or seldom) and inspect their feet by themselves, and only use the app when something seems off: “I keep an eye on them, [...], no need for the app” (Patient 14). This is recognized by the podiatrists, who explain: “What I see a lot, is that people say that [they] used the app, but not anymore because they now check their feet every day, so they don’t think using the app is necessary anymore” (Podiatrist 5).

Some patients experienced important support from their partner in managing their foot health: “I try to really take care of myself, and if I don’t, I still have a lady who’s married to me, and she watches me as well” (Patient 1).

For most patients, inspecting their feet became a part of their daily routine, for instance, in their morning routine (11/24, 46%) or when preparing to go to bed (10/24, 42%). As Patient 1 illustrates: “I usually check in the morning, when I’m putting on my support-stockings, so then I check my feet and grab my phone.” Only 3 (13%) used the app in the afternoon. Most patients used the app in their living room (11/24) or their bedroom (8/24). Only a couple used the app in the bathroom.

While the 9-point checklist is a static question list and worked well for most patients, some patients felt that they could not provide enough information. From the interviews, a patient suggested implementing follow-up questions (eg, branching):

*You’d want follow-up questions, not just the picture. Like, what do you think might be the cause of this? ... Or when there’s callus formation—whether that’s getting worse over time*.

[Patient 1]

##### The Experience of Taking Pictures With the App

In total, 7 patients took a picture after completing the checklist and having answered “yes” to at least 1 question. When a patient was called by the podiatrist after they submitted a picture with a potential issue, they discussed that the picture did not indicate anything serious and felt comforted. To them, this was the main value of the app: “Perfect, this is why I use this app” (Patient 15).

Some patients had issues with taking good pictures and described potential solutions that would help them, such as providing clearer instructions:

*It can be hard to take a good picture of that part of your foot, so instruction would help. Like, keep enough distance and make it concrete, like 10 centimeters or something, and your phone takes the photo or doesn’t when it’s not good*.

[Patient 10]

However, some patients figured out their own solution:

*I put my phone on the ground, put it on selfie mode with a timer of five seconds. Then I put my feet up, and then I have a nice picture of the underside [of my feet].*

[Patient 15]

While all patients could use the checklist themselves, taking a picture was sometimes delegated to their partner:

*... If there are problems, [the App] is good. It’s a tool to help you assess if it’s okay or whether I make a call. If I want to then show something, it’s nice that my wife can take a picture*.

[Patient 10]

One patient found it hard to inspect their feet, so they had support from their partner:

*My wife took a picture. And [she] also inspected my feet and checked everything that was asked. Unfortunately, I am not really capable of doing it myself*.

[Patient 19]

### Theme 2: Podiatrist Experience

#### Podiatrist Perspective on the Value of the App for Their Patients

All podiatrists were positive about their general experience with the system. Most highlight how their patients appreciate the system:

*They really have the idea that someone’s behind it, that really assesses [the picture], which is what they find very nice; to hear that it’s not just filling in an app, but there’s an actual person on the other side looking at the picture*.

[Podiatrist 2]

A frequent problem in developing DFUs is that patients wait too long with an issue before they consult with a podiatrist. Enabling patients to easily share concerns without having to make an appointment is very valuable, as Podiatrist 1 described:

*The positive aspects are that we discovered many problems [though the app], [...], because it’s so easy for the patient. Otherwise, they might have waited for two months before they would call or come over*.

Inversely, podiatrists also like that they can comfort someone who is unnecessarily stressed about a potential issue:

*I really like it, because you can comfort someone because it [appointment] isn’t necessary, when they’re unnecessarily really stressed about it*.

[Podiatrist 2]

While the app was initially developed to support patients remotely during the COVID-19 pandemic, one podiatrist remarked that some patients started using the app when they could not reach the podiatrist physically due to holidays:

*... people are on holiday and use the app because they have developed a wound, and want advice, so [they send in a picture and] we call them. [...] In this way we can give tips remotely, while we can’t see them physically*.

[Podiatrist 2]

#### Inspecting the Feetchecker Images

Some pictures show a clear problem on the foot of the patient. In the picture (Figure 3), the podiatrist recognized a skin tear on 1 toe, causing the podiatrist to flag the patient for follow-up: “Patient should be seen with their shoes/support to prevent worse” (Podiatrist 1).

For other pictures, a podiatrist might recognize potential issues and give the patient a call, as they are interested in the cause of the issue (in this case, the patient had a blue toenail, which can come off). The podiatrist explained:

*What caused this? I need to know the cause and, if necessary, tackle the problem. In the case of one-time trauma, I give an explanation on for how long [the patient] will see the trauma and how the nail will grow*.

[Podiatrist 1]

On one of the images (Figure 3B), the podiatrist recognized a likely corona flair, which is often not a big problem, but might worry patients. Giving the patient a call can comfort a patient, so they do not worry or schedule an unnecessary appointment:

*Usually nothing bad (although it might point to deep venous problems higher up), but nice to give the patient some explanation about this. Anxiety can give a lower quality of life and people tend to worry about this*.

[Podiatrist 1]

Patients sometimes upload unfocused or fuzzy pictures. Nevertheless, podiatrists do become suspicious due to discolorations or other hints, even though the picture is unclear. As Podiatrist 1 said:

*A new picture is necessary, but I already know something here requires attention. Often, we deal with this over the phone to figure out what it is, and how it should be followed up*.

#### Podiatrist Workflow With the Feetchecker App

The podiatrists who inspect the pictures have around 30 minutes a day to do this, but sometimes this is too short for the number of pictures and phone calls. Prioritizing cases costs extra time they do not always have:

*What I find difficult is that I have half an hour to go through a list of around 15 pictures, and I don’t yet know which patient photographed a mole, and which one a large wound. So, I have to actively open the pictures and filter on importance and priority, which costs time*.

[Podiatrist 1]

Some podiatrists who inspect the pictures work together with the clinic’s secretaries, to make the appointments, but these podiatrists also felt the need to inform their patients via mail as well: “... I do send a short mail to people, to say that our secretary will contact them, because I want to see them a bit quicker” (Podiatrist 2).

In the backend of the system, podiatrists have no access to use statistics for individual patients, only if a patient sends a picture. One podiatrist remarked that they would like to keep an eye on that, as it gives them an idea about how patients inspect their feet:

*I don’t have insights into how the app is used, are they really going to use it? I can ask or repeat, but in the end, the question is how they’re going to adopt it*.

[Podiatrist 3]

### Theme 3: Instruction and Education Toward Using the App

#### Instructions on How to Use the Feetchecker App

When podiatrists recommend the app to their patients, they focus on getting patients to get to know their own feet, as Podiatrist 5 instructed their patient: “Use [the app] a couple of days so you know what to pay attention to, and that you get to know your own feet, and use that to conduct foot screenings.”

We observed a variety in how podiatrists instructed the patients; some recommended to use the app frequently, while others focused more on using the app when patients had doubts about an issue with their feet: “If you doubt about something, use it, because then you’ll know for sure” (Podiatrist 5).

Most patients started using the app because the podiatrist introduced them to the app and motivated them to use it. However, for some patients, family played a big role in adopting the app, as Patient 1 illustrates: “I had to laugh at first, but my son and daughter-in-law are both doctors, so they said that they were also dependent on what their patients told them, so I do [use the app].”

Podiatrists also mentioned that they often help install the app during a consult and show how it is done on their own phone: “When I can, I’ll let [people] install it here or have the accompanying family member install it” (Podiatrist 4).

While the podiatrists included in this study were enthusiastic to recommend the app to their patients, one podiatrist remarked that that sentiment is not yet prevalent for the majority of podiatrists. Over recent years, health care professionals have had to deal with an increasingly growing burden of using digital systems to document every task. Not everyone is therefore happy to add more of such tasks to their job:

*But most podiatrists are not as motivated to recommend the app, because they don’t really see it as a “present” to patients. Because [they see it as] just more information [they need to deal with]*.

[Podiatrist 1]

This is also a reason that podiatrists did not always update the system with whether a patient was followed up after a Feetchecker picture was submitted when an appointment was already planned anyway.

#### Patient Education Through the Feetchecker App

Functionalities targeting patient education seem to improve patient awareness and understanding of what to look for when inspecting their feet: “It motivates you to really check your feet, and you know better what to look for” (Patient 6). The app has knowledge clips (not all clips were available at the time of the study) as a form of providing information to patients beyond the consultation, which some patients appreciated: “The knowledge clips are very useful. Too bad not all of them are [active] yet” (Patient 4). Yet, for most patients, these clips were considered an “extra,” as Patient 9 says: “The knowledge clips are nice extras, but it’s (of course) very important to just get into contact with a podiatrist very quickly and effectively.”

Nonetheless, the educational features were described as important, as a podiatrist highlighted the importance of providing information beyond the consultation, since they felt like many patients would forget or misremember information they provided:

*We just want to know what they can do themselves. Of course, we already teach them a lot in our consultation, but it’s nice if they can read it again, at their own tempo*.

[Podiatrist 5]

Additional information beyond just the feet was also indicated as a desired feature of the app: “You shouldn’t only look at your feet, but also the lower leg, so some information on that would be a good addition” (Patient 10).

### Theme 4: Challenges in Accessibility, Digital Skills, and the Necessity of Checking Feet

#### Barriers Toward Adopting Digital Care Apps

From our interviews and the use data, we observed that not every patient had the self-management capacity to keep using the app over time. While the podiatrists informed and supported patients in adopting the Feetchecker app, they noted that not everyone is interested in keeping an eye on their own foot health:

*[...] With another patient, during intake we discuss the app, and they mention they don’t want it, as they feel it’s enough already and they don’t want to go through a check every week ... From these remarks, I can tell to what extent I can ask them to self-manage*.

[Podiatrist 3]

Others mention that some patients feel it as an “obligation” to use the app, and therefore, reject it, as one podiatrist said: “You’ll meet some people who are really hesitant, and don’t like using another app, because they feel it’s an obligation” (Podiatrist 2). Furthermore, the seriousness of DFUs does not always get through to patients, making them doubt the importance of frequently checking their feet: “But there’s also people who don’t really understand why it’s important to really check their feet every day” (Podiatrist 5).

#### Accessibility and Digital Skills

Podiatrists noticed that the Feetchecker app is not accessible to patients who are not proficient in the language of the app (Dutch): “A negative point is that [the app] us currently not facilitating people from a different background or people who aren’t proficient in the language of the app” (Podiatrist 1). Often, these patients bring a relative along to a consult who can help translate the advice from the podiatrist. Podiatrists also explained the app to them, hoping that the relative helps the patient understand the necessity of frequently checking the health of their feet: “The biggest hope you have, is that the child translates it, sits close and maybe take that picture if they think it’s not going well” (Podiatrist 4).

Sometimes, patients have trouble making clear pictures that show a potential issue, making it hard for the podiatrist to assess the problem:

*Sometimes you get a picture where I’m not sure whether I’m looking at their foot or their lower leg. So it can depend on how they’re making the picture in the Feetchecker App*.

[Podiatrist 2]

One patient also mentioned having difficulties in checking picture quality because the app does not show a “preview” of the picture after it has been taken: “I can’t see whether the picture is good” (Patient 22).

## Discussion

### Principal Findings

This paper describes the results of a 3-month mixed methods evaluation study of the Feetchecker app with 24 patients with type 2 diabetes and at risk of DFU. We examined app usefulness, engagement, and overall user experience through a synthesis of quantitative and qualitative data.

Overall, this study found that the majority of patients perceived self-monitoring the health of their feet with Feetchecker as useful and empowering. This study showed that the app helped most patients play a more active role in checking for potential problems, and patients saw real value in sharing the Feetchecker pictures with podiatrists. Previous efforts in using pictures showed that people perceived this form of self-monitoring as valuable [19]. This study contributes to the literature by identifying an effective and accessible way of self-monitoring potential foot problems in people’s everyday routines, focusing on at-home use. The results showed that most patients frequently conducted feetchecks in their own home and recognized that the accessibility of a specialized podiatrist through an easy-to-use app was useful. While the majority of patients did have a look at the timeline and information features in the app, most did not actively use these over longer periods of time.

While engagement in using eHealth apps often drops off after the first week [32,33], we observed good retention over time and believe that most patients had effective engagement with the Feetchecker app; they were able to use the app in a way that fits their needs. Analysis of data logs from the app identified 3 main engagement patterns with the app: continuous, frequent, and no to little engagement. While some patients stopped their use of the app altogether, for others, a period of low use perhaps does not indicate “engagement failure,” as Ploderer et al [19] describe, but rather, a sign of changing needs over time, which is more in line with the conceptualization of effective engagement by Yardley et al [26]. How patients were instructed in using the app by their podiatrists is likely to be important to the level of engagement. Kilic and Karadağ [21] asked patients to use the app every time they measured blood glucose and send foot observations daily. Ploderer et al [19] asked patients to use MyFootCare each time they changed their wound dressing away from the clinic to introduce a routine to the task. In our study, podiatrists were free to recommend how patients should engage with the app. In practice, podiatrists often promoted steady checking routines (eg, every week) that fit patients’ needs and routines. We found that podiatrists did not mind that patients used the app less after a while. As patients learned how to inspect their own feet by using the app and then would keep doing so regularly (without the app), podiatrists understood that patients would use the app when necessary.

Research on promoting self-management practices for patients with diabetes showed that just handing patients flyers or a website is insufficient for engaging them in the intervention [34,35]. In our study, we find that having a podiatrist help explain the app in person and follow up if something is not right can help with starting and continuing use of the Feetchecker app.

In our results, we found that some patients experienced the picture-taking process as somewhat difficult due to either physical limitations or their digital skills. This sometimes results in blurry, under- or overexposed pictures. Potential solutions to this are shared in work from Anthony et al [36], who saw the best results in picture-taking by using tools such as a selfie-stick or asking another adult to help. However, Kolltveit et al [15] share that despite having health care professionals take the pictures, they found that a lack of space, light, and a purposeful working position seemed to impede the use of the software. Similarly, Swerdlow et al [20] showed promising results from their “Foot Selfie” system, but this required a specialized tool. Since all these systems have promising advantages, as well as disadvantages, we argue for a more deliberate approach that tailors the choice of intervention to the patients’ needs and capabilities. Furthermore, the necessary quality of pictures depends on the intended use. Pictures for monitoring wound healing might need to have a good and clear quality (eg, for the algorithm to identify edges), while a trained podiatrist can already use a simple homemade picture with lesser quality to judge whether a patient should be seen or not.

### Limitations

This study was based on a mixed methods evaluation with 24 patients and 5 podiatrists, which limits the conclusions we can draw on the effects of this intervention on actual long-term prevention of DFU. The quantitative results are used descriptively, as this study design was not intended to provide results on clinical outcomes or effects on DFU formation. Our strengths are in real-life deployment for 3 months in everyday situations, leading to high ecological validity. This was highly informative of app engagement, use cases, types of users that emerge, and how patients used the app in their everyday life.

While we did not observe any technical problems with the Feetchecker app system, one patient did not enter their patient number correctly in the app, which makes it impossible to link the output of the app to the EHR. For future work, we aim to work on more robust authentication methods, as well as providing patients with a leaflet where the podiatrist can write down the patient number.

While a big benefit of the Feetchecker app is the ability for remote monitoring by podiatrists through submitted pictures, the app has no 2-way communication. If a podiatrist wants to ask for a new picture, they need to call the patient. We aim to investigate the potential to use 2-way communication features to reduce unnecessary calls or visits to the clinic.

### Future Perspectives

Many people with or at risk of DFU have difficulties with the usability of mobile apps [37]. Future studies should focus on improving the usability of the Feetchecker app to be more inclusive toward patients with visual impairments and limitations of physical mobility and should offer multiple language options. As Mannheim et al [38] recommend, future work should include this target group in the design process. Furthermore, Wang et al [39] suggest to further investigate optimal formats and the frequency of engaging patients as well as build better tailoring of reminders.

Similar to skin cancer screening apps such as Skinvision [40], we envision that the picture-taking feature of the Feetchecker app might be enhanced through the use of algorithms that can recognize whether an image is of sufficient quality for effective inspection by the podiatrist. Recent research has shown potential in using artificial intelligence systems to support triage or diagnostics [41]; however, these systems are not accurate enough to be implemented in real-world use [42,43]. While we see the advances of artificial intelligence–powered screening systems [44-46], we must not lose sight of having a human-in-the-loop to make sure people are being listened to, as we showed that this human connection was a primary positive result of this study. Future studies will also be needed to determine if the Feetchecker app can reduce the need for in-person clinic visits, the incidence of severe or infected DFUs, and eventually reduce the cost of medical care.

### Conclusions

The Feetchecker app supported patients in self-monitoring risk factors associated with DFU through routine checks and quick contact with a health care professional in case of a potential issue. Patients generally described a positive user experience and considered the app helpful. While mHealth tools are not for everyone, user engagement for many patients was high and shows that such apps can offer support for patients able to use them. Furthermore, podiatrists appreciated how the pictures enabled accessible and quick contact with patients. Supporting health care professionals in integrating mHealth tools in their regular care is important toward adoption and sustained use. Future work needs to be done to better understand how mHealth can be leveraged to better integrate into the everyday routines of patients at risk of DFU. This is crucial to promoting self-monitoring of foot health, as it may shift the focus from managing wounds to effectively preventing the need for care in the first place. Innovative solutions like these are urgently needed to enhance current systems for the prevention and care of diabetic foot ulceration in a world where the number of patients with diabetes is increasing quickly.

## Supplementary material

10.2196/80769 Multimedia Appendix 1 Feetchecker app questions.

10.2196/80769 Multimedia Appendix 2 Feetchecker app description.

10.2196/80769 Multimedia Appendix 3 Care profile description.

10.2196/80769 Multimedia Appendix 4 Feetchecker images and related descriptions.

10.2196/80769 Multimedia Appendix 5 Feetchecker surveys.

10.2196/80769 Multimedia Appendix 6 Feetchecker interview guides.

10.2196/80769 Multimedia Appendix 7 Demographics per participant.

## Abbreviations

DFU: diabetic foot ulcer
EHR: electronic health record
mHealth: mobile health
