# Supplementary material for: Self-Monitoring Risk Factors for Diabetic Foot Ulceration With the Feetchecker App: Mixed Methods Study
Source: JMIR Form Res. 2026 May 27;10:e80769. doi: 10.2196/80769 (PMC13215667; doi:10.2196/80769)
Supplement: Multimedia Appendix 1 [file formative-v10-e80769-s001.docx]

**Appendix 1.** Feetchecker App Questions in the Check.

Translated from Dutch to English.

1. Do you have soft (white) skin between your toes?
2. Do you have red spots on your feet?
3. Do you have red spots on your feet or toes that begin or get worse while wearing your shoes?
4. Do you have calluses with a dark discoloration on your foot?
5. Do you see a blue discoloration underneath your toenails?
6. Do you see other discoloration (different from your skin color) on your foot?
7. Do you have a wound or small wound on your foot?
8. Is your foot red, thick and swollen?
9. Is on of your feet warmer or colder than the other foot?
