# Supplementary material for: Self-Monitoring Risk Factors for Diabetic Foot Ulceration With the Feetchecker App: Mixed Methods Study
Source: JMIR Form Res. 2026 May 27;10:e80769. doi: 10.2196/80769 (PMC13215667; doi:10.2196/80769)
Supplement: Multimedia Appendix 2 [file formative-v10-e80769-s002.docx]

**Appendix 2. Description of Feetchecker App features.**

The application can be downloaded in the Apple Appstore: <https://apps.apple.com/nl/app/diabetes-voetencheck/id1504085498>

or Google Playstore: <https://play.google.com/store/apps/details?id=nl.stofloos.zelfcheck&hl=nl&gl=US>

More information can be found on the Application website: <https://diabetesvoetencheck.nl/>

Podiatrists recommend the Feetchecker App to patients, often during their first or second consultation. The podiatrist explains the purpose and use of the application and usually recommends patients to use it once of twice a week, or, however they see fit. They also show the instructive videos and relevant information. Patients often use the Feetchecker app as a part of their routines (e.g. bathroom in the morning), usually after a notification goes off. They launch the app, go through the ‘check-list (feature 1), and take a picture if prompted by the app (when they have answered ‘yes’ on one or more questions.

A number of Podiatrists at Rondom Podiatrists have a ~30-minute timeslot a week to check new pictures in the EHR system. The guideline is that they always follow-up, usually by asking the clinic secretariat to make an appointment, or by calling themselves to explain / ask details and discuss whether an appointment is necessary.

The application contains the following main functions:

#### Features

1. **The Feetcheck**. The Feetcheck is a question list of 9 yes/no questions such as “Do you see red spots?” or “Do you see any cuts?” (Appendix 1) (Figure 1c). Users can do the ‘long’ version, in which every question is on a separate screen with a yes/no button, or a ‘short’ version, where all questions are placed on a single screen and allows answering yes/no to all questions in one press. All questions are guided by example pictures to improve understanding and accuracy.
2. **Picture Taking.** If users answer one of the Feetcheck questions with a “yes”, they are prompted to take a picture of the relevant part of their foot (Figure 1f). The function allows the user to take multiple pictures, retake the picture, or select a picture from the gallery. The picture is then uploaded to the system of the podiatry clinic and inspected by a podiatrist specialized in diabetic feet.
3. **Timeline.** Users can review their previous Feetchecks and pictures in a chronological timeline view (Figure 1d). Users can use this feature to compare their pictures over time, or to show the pictures to the podiatrist during a consultation.
4. **Knowledge Clips**. A collection of curated knowledge clips, created by the expert diabetes podiatrists of the clinic to offer information and advice on a variety of topics (Figure 1e). This information follows the International Working Group on the Diabetic Foot (IWGDF) guidelines. Topics include diabetes, diabetic foot, footcare, footwear use, neuropathy, vascular problems, and more. If users have finished watching a video, they are presented with a relevant multiple-choice question on the video's topic to check if they understood the information.
5. **Notifications**. Users can set custom notifications to remind them to conduct a Feetcheck (Figure 1b). They can set the frequency, day of the week and the time.
6. **Points system**. Users can collect points by performing Feetchecks, upload pictures, watch videos and answer knowledge questions. These points can earn users' badges. In the current design of the Feetchecker App, no tangible rewards are connected to achieving badges. This function acted as a first exploration in a point system to incentivize use of the app.
