# Supplementary material for: Self-Monitoring Risk Factors for Diabetic Foot Ulceration With the Feetchecker App: Mixed Methods Study
Source: JMIR Form Res. 2026 May 27;10:e80769. doi: 10.2196/80769 (PMC13215667; doi:10.2196/80769)
Supplement: Multimedia Appendix 3 [file formative-v10-e80769-s003.docx]

**Appendix 3 – Podiatric Care in the Netherlands and Care Profile Descriptions.**

In the Netherlands podiatric care follows a specific care pathway, which can be different from how other countries have implemented care for people with, or at risk of, DFU.

Often, patients that receive a Diabetes diagnosis from their physician receive a referral to the podiatrist. The podiatrist will then do an intake consultation and assess risks and provide information on maintaining foot health, based on the care needs of the patient. Podiatrists in the Netherlands conduct clinical tasks such as diagnosis and treatment plans (and are certified to do so). For more information on how responsibilities between healthcare professionals in The Netherlands are distributed, we refer to the Dutch Regulations on foot care for diabetic feet [1].

In the Dutch healthcare regulations [1], patients with diabetes will be classified by their physician, or other healthcare professional, with a Sims [2] risk classification. This clinical classification is an internationally accepted system, used by The International Working Group on the Diabetic Foot (IWGDF) [3] for assessing diabetic foot risks and often conducted by the podiatrist. The podiatrist will then assess which Care Profile (“Zorg Profiel”, or abbreviated: “ZP”) the patient falls under. A care profile indicates which ‘care package’ is needed to limit the risk of developing foot wounds. Based on the care profile, the necessary foot care is organized for the patient, and the reimbursement for this is also determined. There are four care profiles in total. The care profiles are structured as follows:

A Sims 0 classification gives a **Care profile 0**, A Sims 1 classification leads to **Care profile 1.** A Sims 2 classification and the diagnosis of decreased sensation in the feet and reduced circulation leads to **Care profile 2**. The Sims 2 classification and the diagnosis of decreased sensation in the feet, reduced circulation, *and* pressure sores leads to **Care profile 3.** The Sims 3 classification and the presence of a previous foot wound or amputation, or this is a case of inactive Charcot foot or renal replacement therapy (dialysis), leads to **Care profile 4.**

This information is of use in the research we conduct, as we can use this to include or exclude patients in our studies. Furthermore, patients with previous problems with their feet might understand the use of a preventative app better, or might have already received instructions for checking their feet more often.

[1] Uitvoering voetcontrole Diabetische Voet. 2017. De Richtlijnen Database. Federatie Medisch Specialisten. <https://richtlijnendatabase.nl/richtlijn/diabetische_voet/preventie/uitvoering_voetcontrole.html>

[2] David S. Sims, Peter R. Cavanagh, Jan S. Ulbrecht, Risk Factors in the Diabetic Foot: Recognition and Management, *Physical Therapy*, Volume 68, Issue 12, 1 December 1988, Pages 1887–1902, <https://doi.org/10.1093/ptj/68.12.1887>

​[3] IWGDF. (2023). *IWGDF Guidelines (2023 update)*. Www.Iwgdfguidelines.Org. https://iwgdfguidelines.org/guidelines-2023/
