# Supplementary figures and images for: Self-Monitoring Risk Factors for Diabetic Foot Ulceration With the Feetchecker App: Mixed Methods Study
Source: JMIR Form Res. 2026 May 27;10:e80769. doi: 10.2196/80769 (PMC13215667; doi:10.2196/80769)

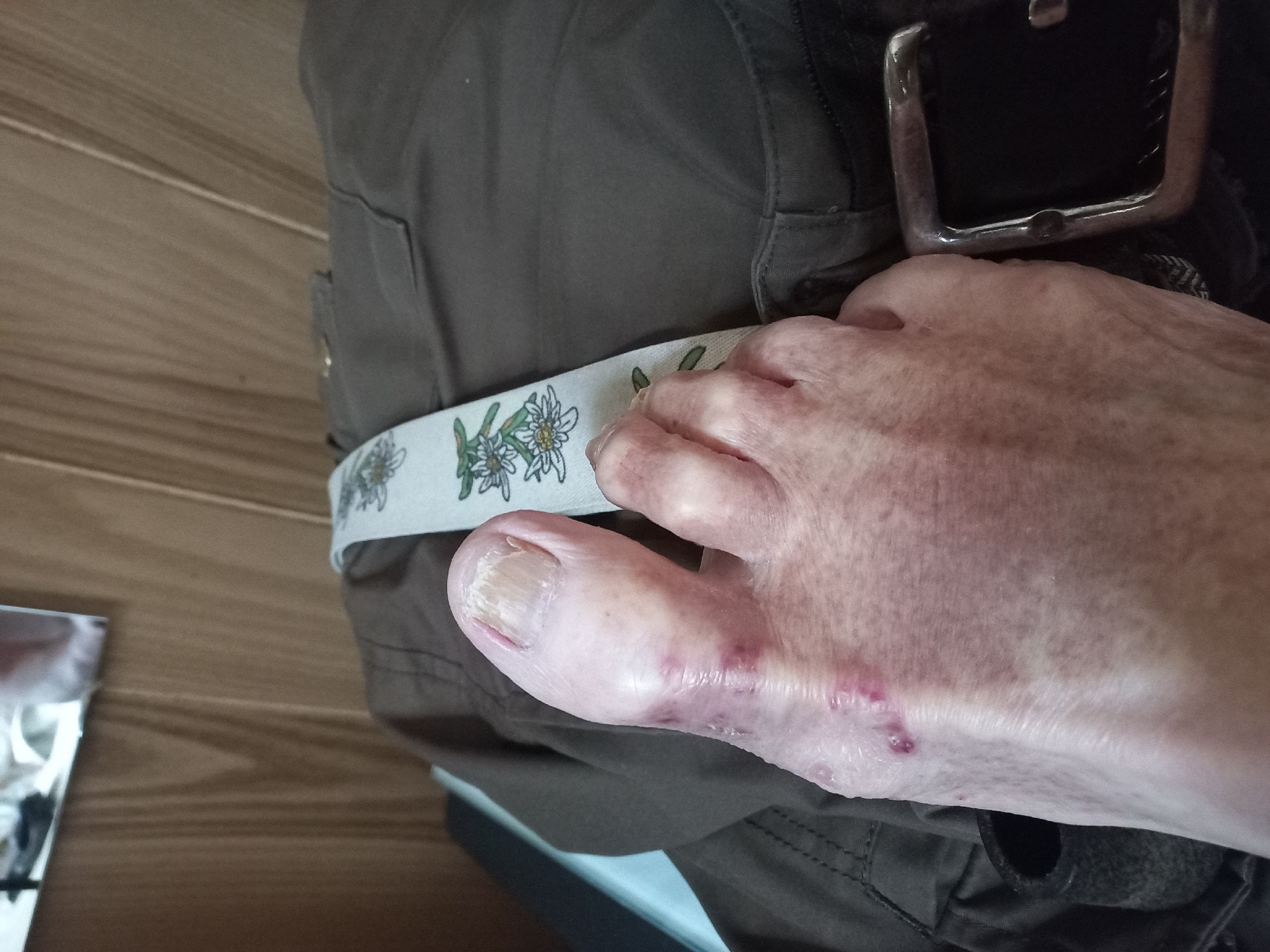

Supplement: Multimedia Appendix 4 [file formative-v10-e80769-s004.zip › Figure 3e.jpeg]

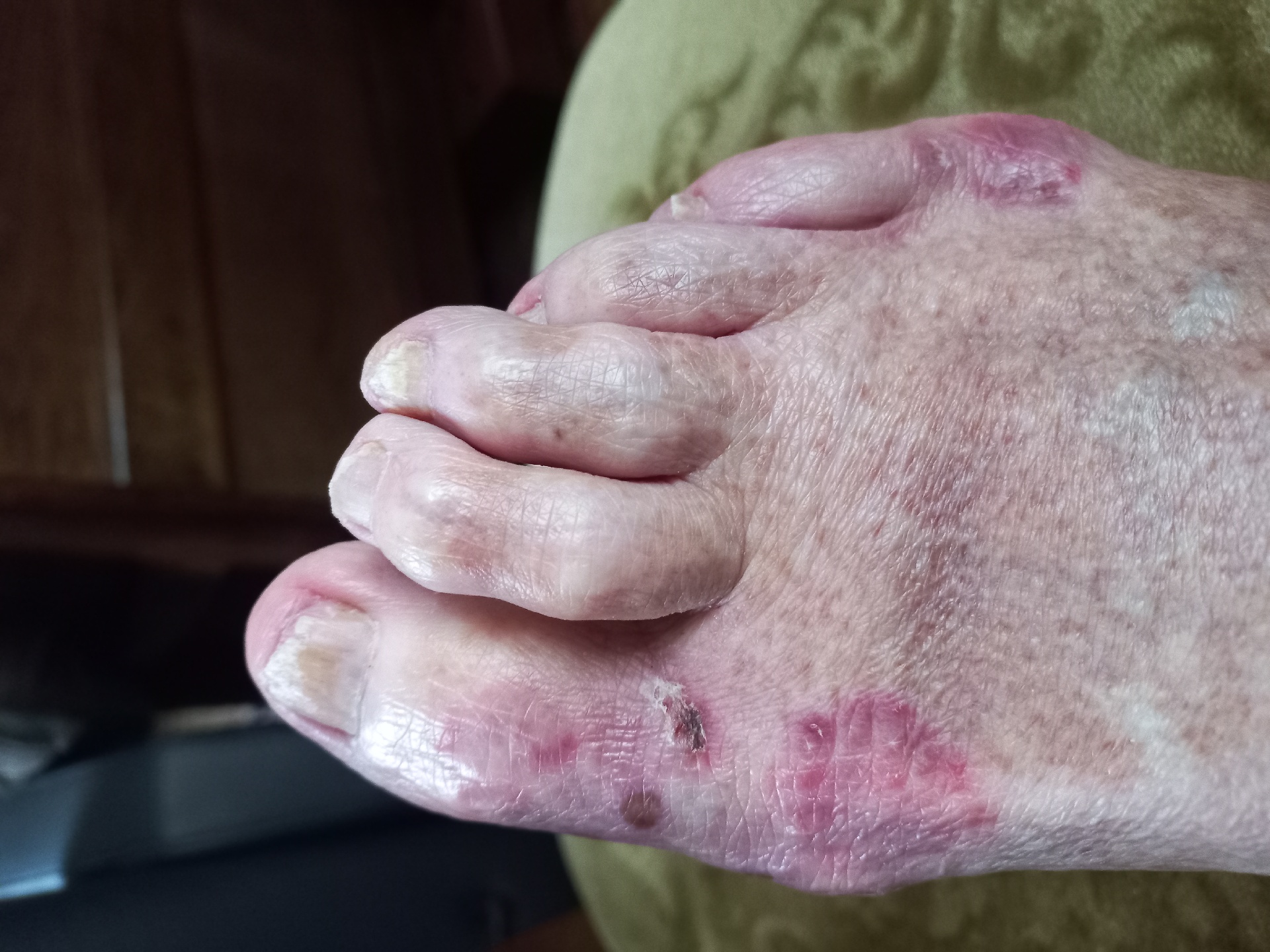

Supplement: Multimedia Appendix 4 [file formative-v10-e80769-s004.zip › Figure 3f.jpeg]

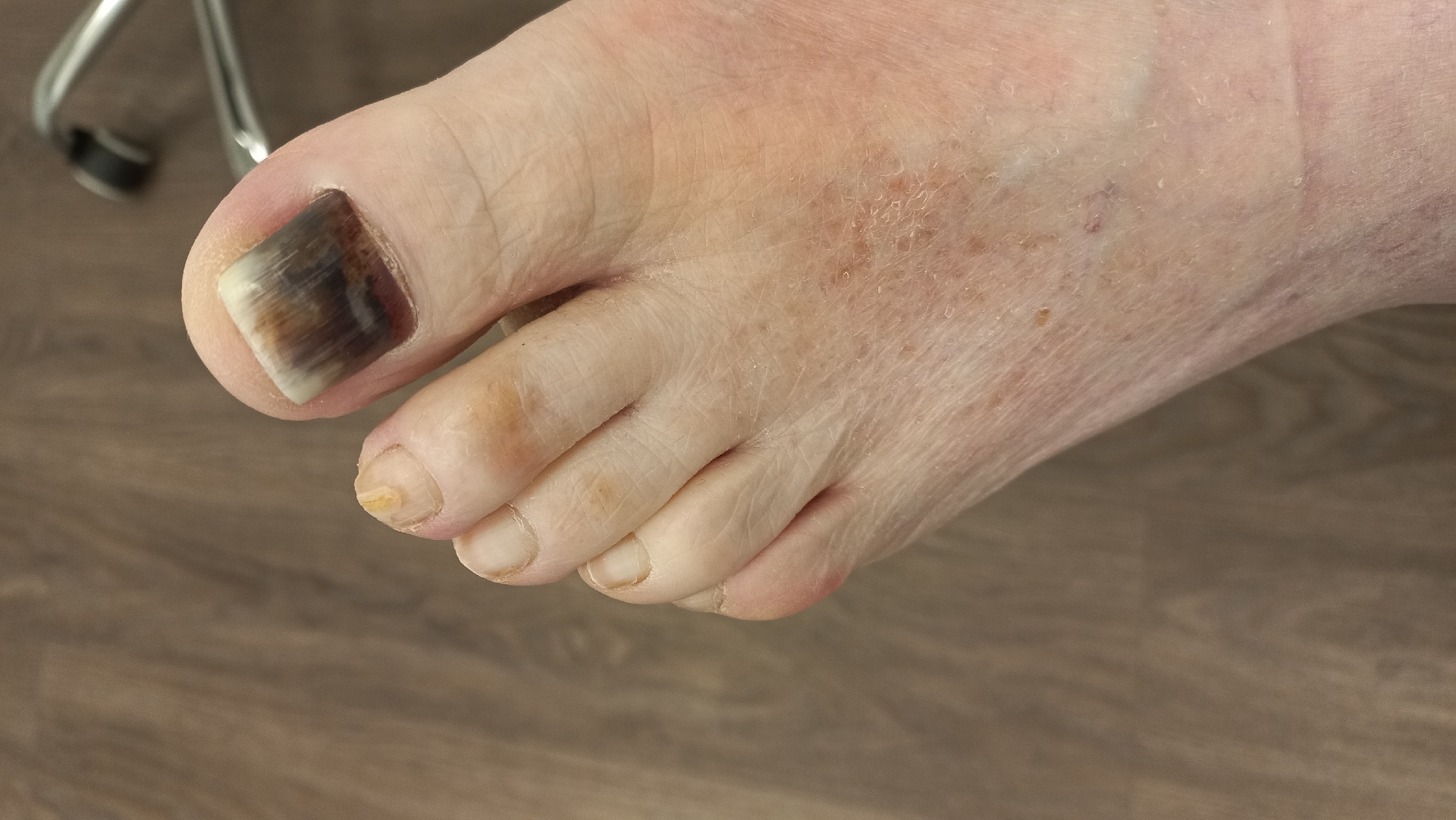

Supplement: Multimedia Appendix 4 [file formative-v10-e80769-s004.zip › Figure 3g.jpeg]

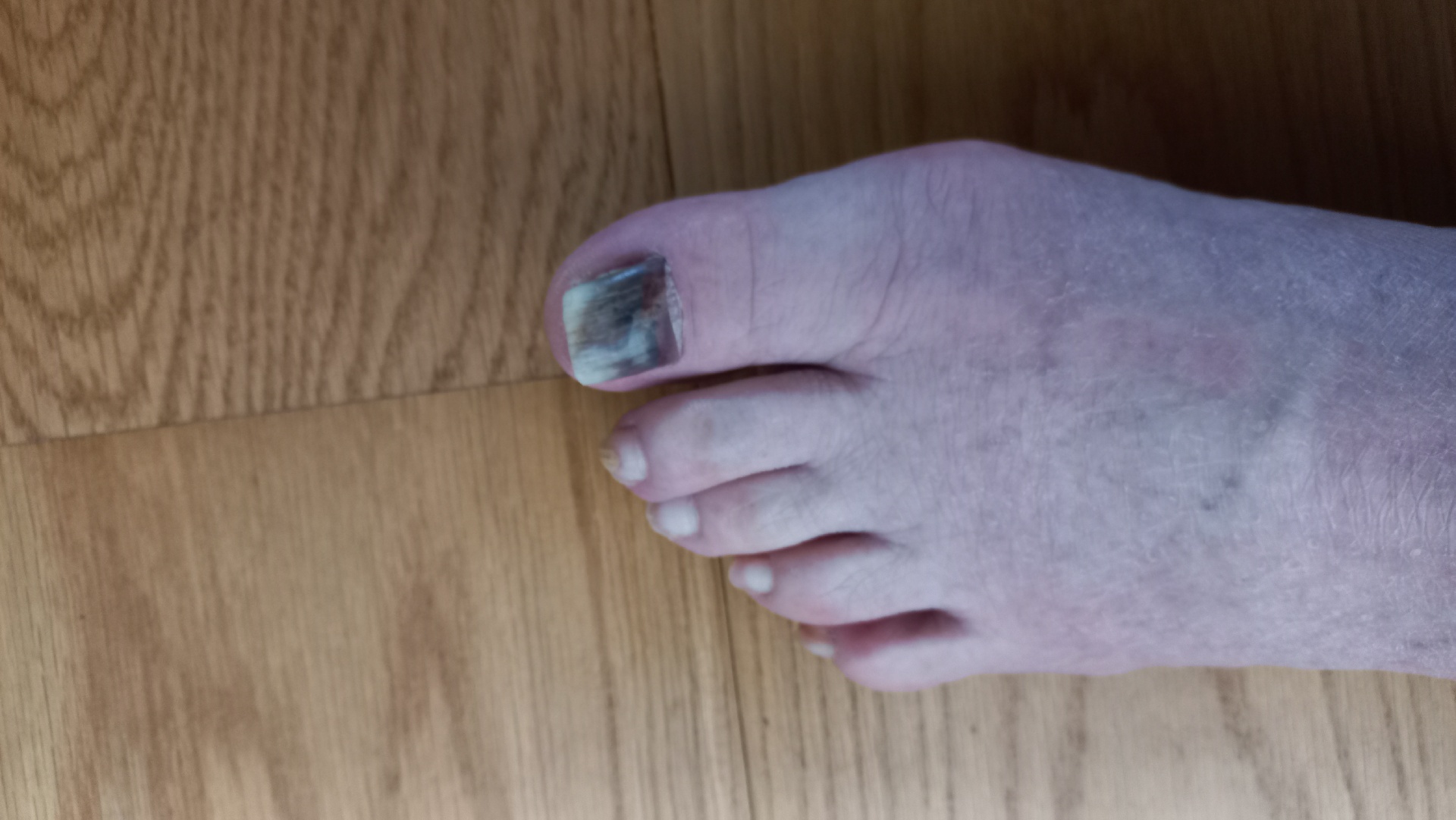

Supplement: Multimedia Appendix 4 [file formative-v10-e80769-s004.zip › Figure 3h.jpeg]

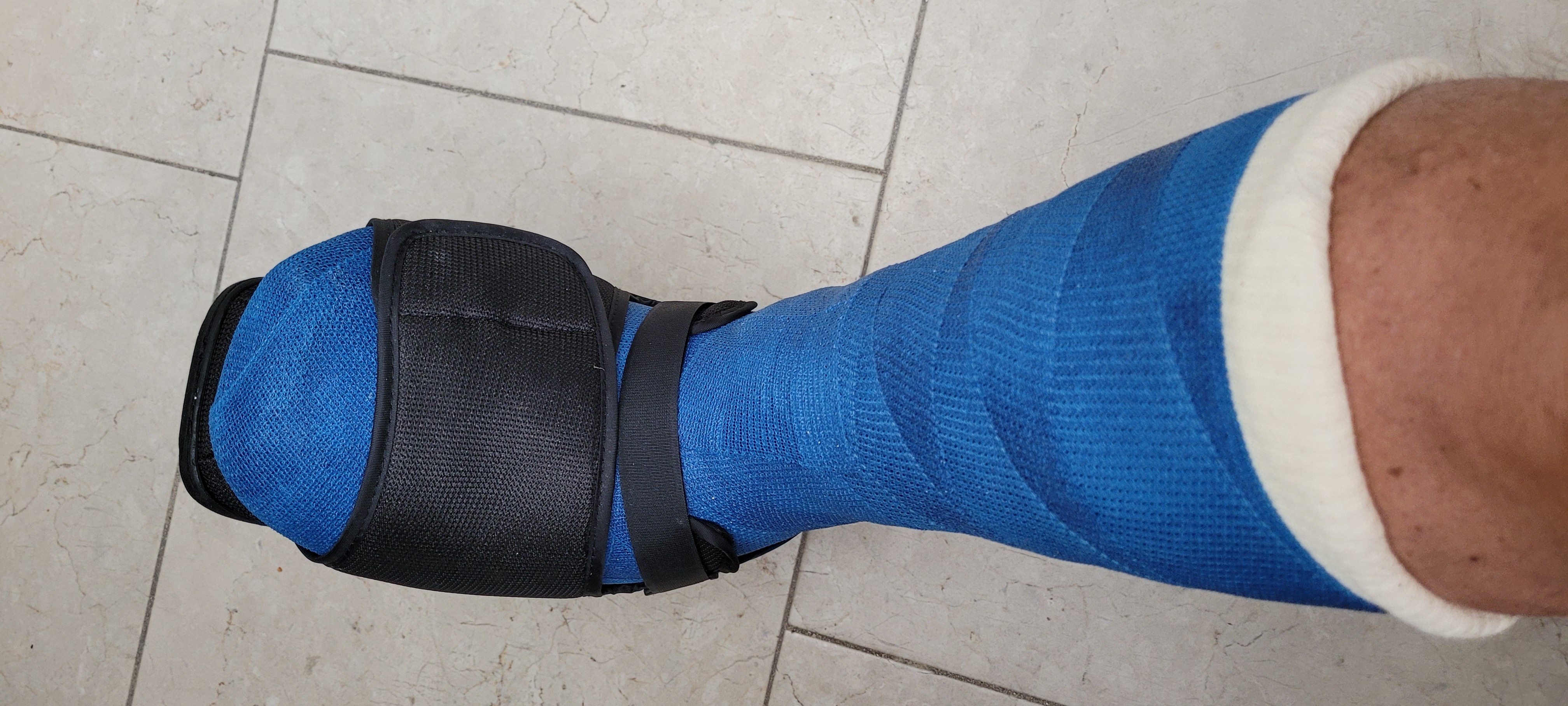

Supplement: Multimedia Appendix 4 [file formative-v10-e80769-s004.zip › Figure 3i.jpeg]

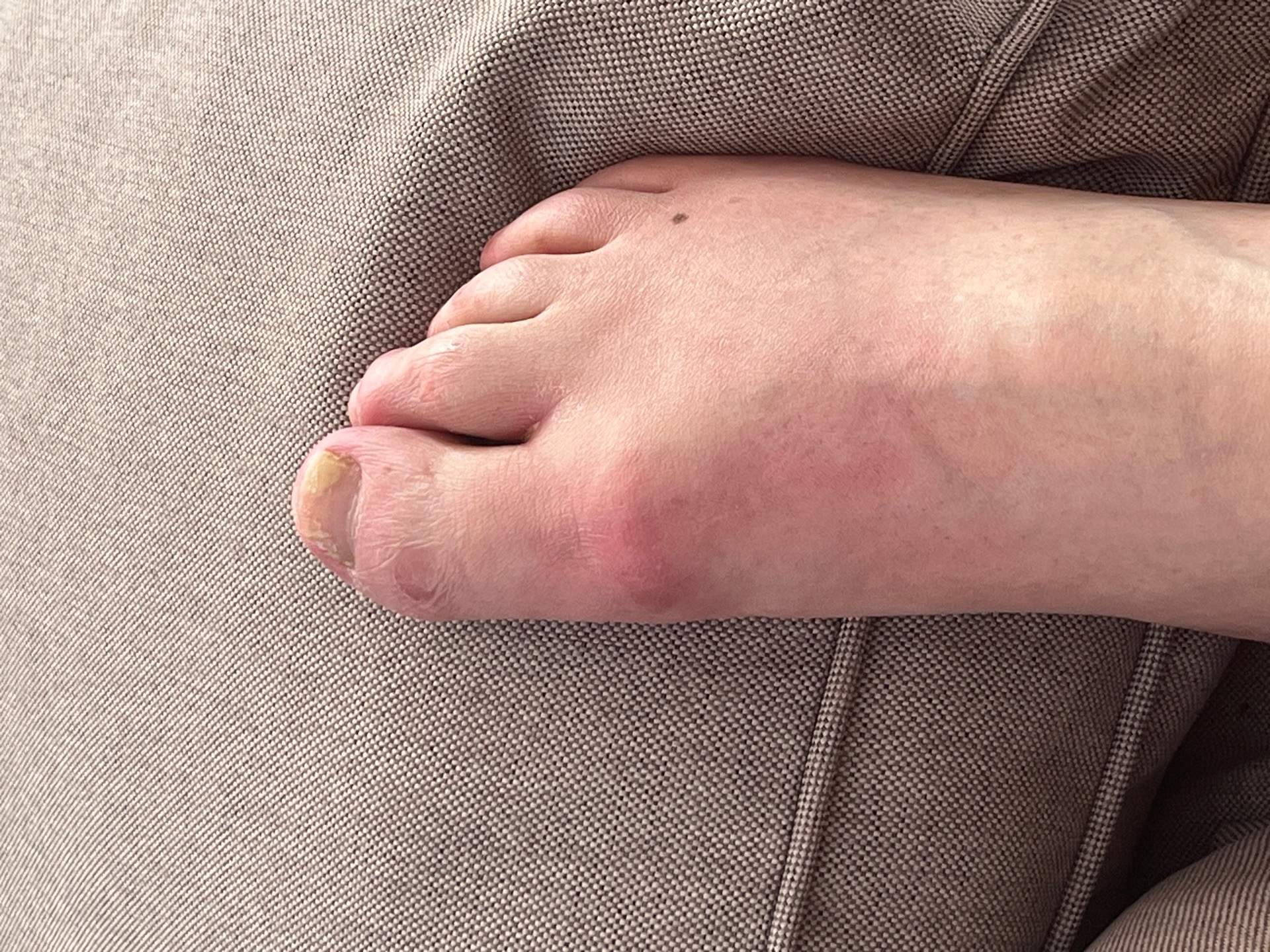

Supplement: Multimedia Appendix 4 [file formative-v10-e80769-s004.zip › Figure 3a.jpeg]

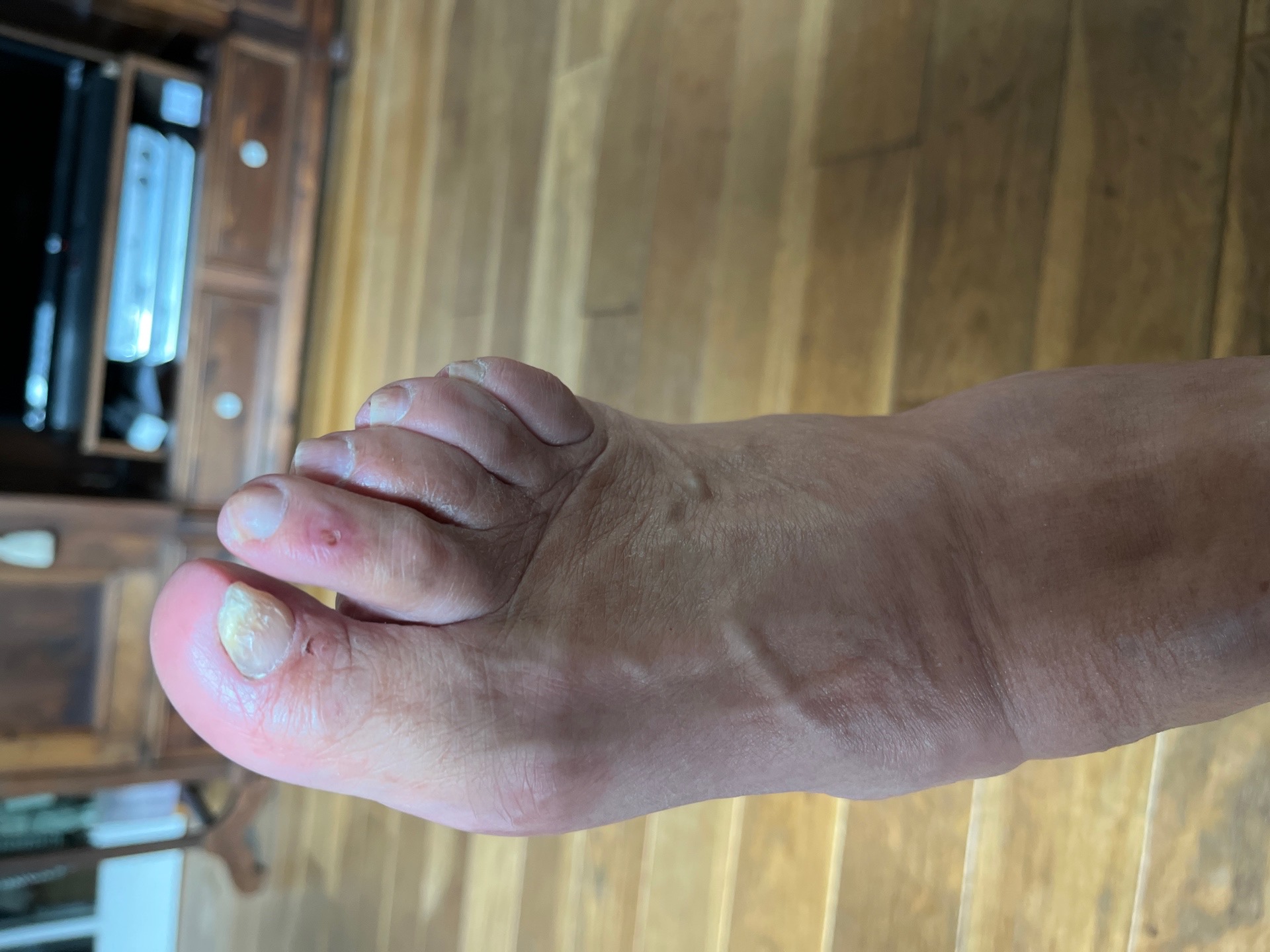

Supplement: Multimedia Appendix 4 [file formative-v10-e80769-s004.zip › Figure 3b.jpeg]

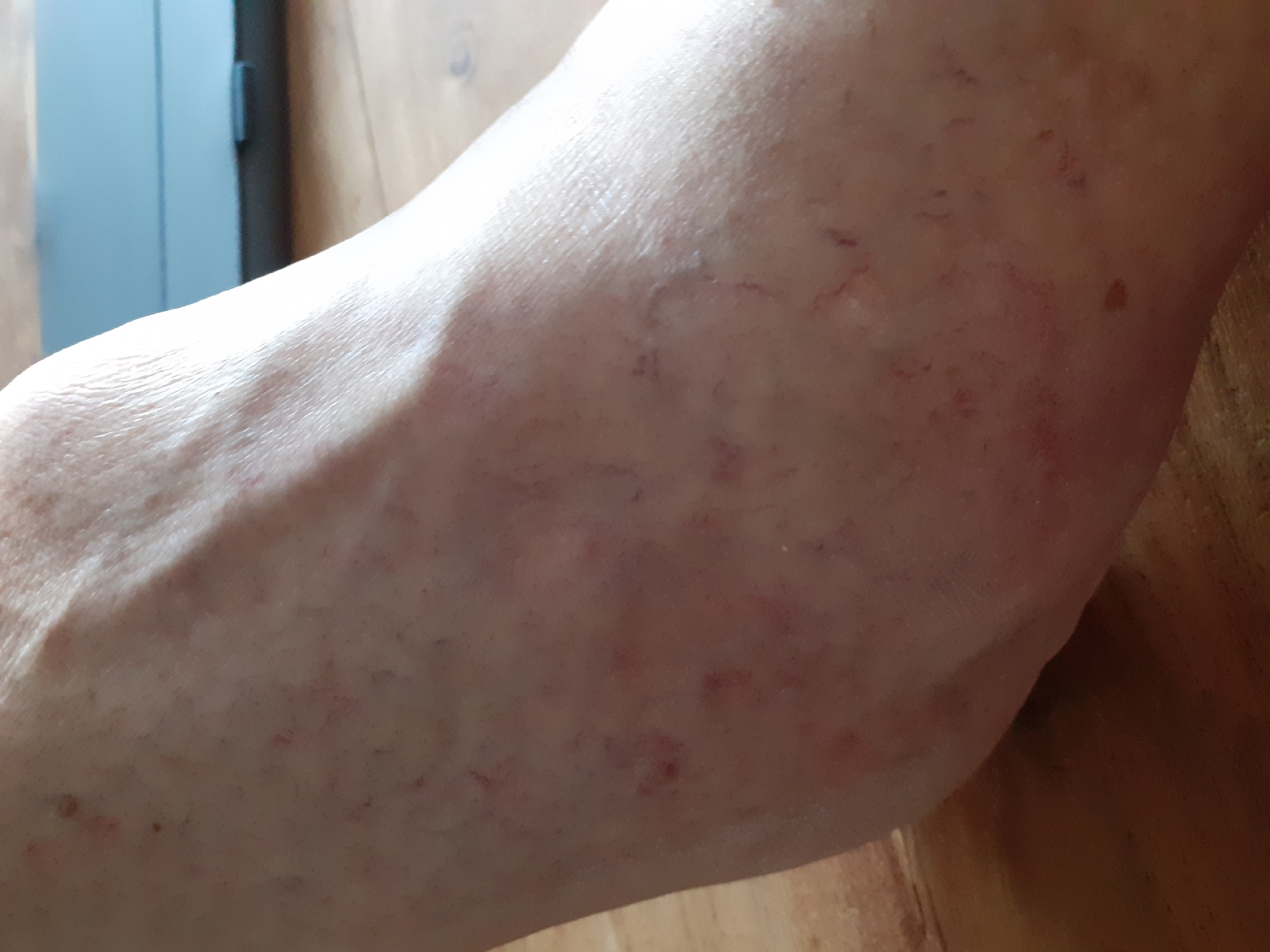

Supplement: Multimedia Appendix 4 [file formative-v10-e80769-s004.zip › Figure 3c.jpeg]

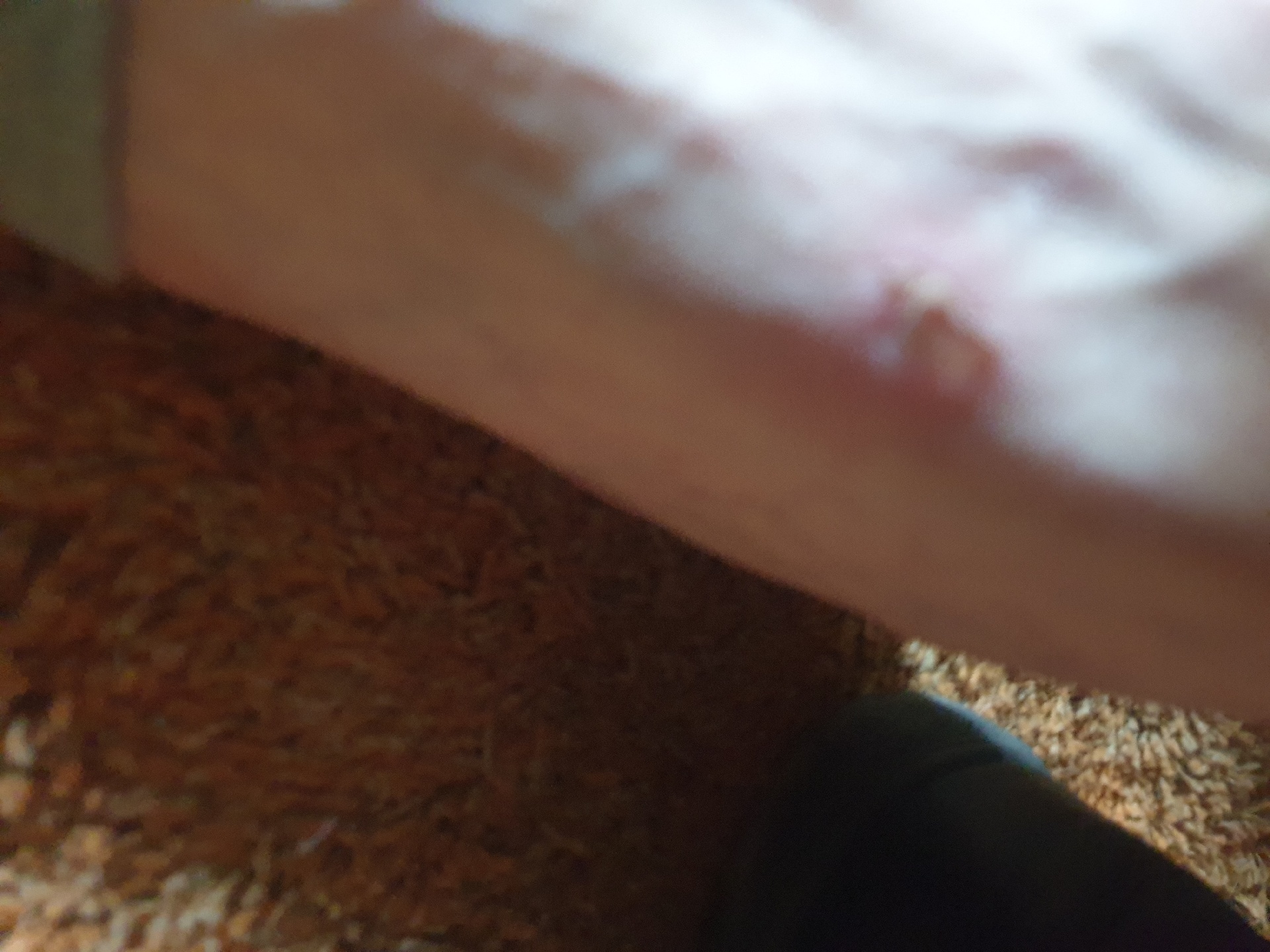

Supplement: Multimedia Appendix 4 [file formative-v10-e80769-s004.zip › Figure 3d.JPEG]
