# Supplementary material for: Self-Monitoring Risk Factors for Diabetic Foot Ulceration With the Feetchecker App: Mixed Methods Study
Source: JMIR Form Res. 2026 May 27;10:e80769. doi: 10.2196/80769 (PMC13215667; doi:10.2196/80769)
Supplement: Multimedia Appendix 5 [file formative-v10-e80769-s005.docx]

Patient Feetchecker Intake Survey.

1. Do you use the Feetchecker App? And if so, for how long?
2. What is your Patient Number?
3. What do you expect the Feetchecker App to do for you?
4. How long have you (already) used the Feetchecker App?
5. Have you used the Feetchecker App before?
6. How did you come into contact with the Feetchecker App?
7. How often do you use the Feetchecker App?
8. What do you think are the most valuable functions of the foot check app with regard to checking your feet?
9. To what extent have you used the Feetchecker App?
10. Do you still use the Feetcheck App?
11. If you stopped using it, why did you stop using the Feetchecker App?
12. Do you ever check your feet for wounds (or have them checked) outside of healthcare consultations?
13. How often do you check your feet for wounds?
14. How do you check your feet for wounds?
15. Do you have experience with receiving care via digital means, such as a website, app or digital watch?
16. What do you expect the Voetencheck app to do for you?

Patient Feetchecker App Post Survey

1. What is your patient number?
2. Do you still use the Foot Check app? If not, why did you stop using the Foot Check App?
3. How often have you used the Foot Check App recently?
4. Which functions of the Foot Check App have you used?
5. Why did you like these functions?
6. Which function(s) of the Foot Check App have you not or hardly used? (multiple answers possible)
7. Are there aspects of the Foot Check App that you find difficult or confusing to use? If so, which ones and can you briefly explain why? (you may name multiple aspects)
8. To what extent has using the Foot Check App helped you check your feet?
9. Why has the Foot Check App been able to help you check your feet or not?
10. To what extent has the Foot Check App made you aware of potential risks and complications associated with diabetic foot problems? If so, in what way?
11. At what time and place do you most often use the Foot Check App?
12. To what extent are you satisfied with the available information and knowledge clips within the Foot Check App regarding checking your feet and preventing diabetic foot problems?
13. What do you think are the most pleasant function(s) of the Foot Check App? (multiple answers possible)
14. Are you missing information and knowledge clips (regarding diabetic foot problems) and would you like to see them back in the Foot Check App?
15. What do you think are the most valuable functions of the Foot Check App regarding checking your feet?
16. At which location do you usually use the Foot Check App? (For example; Living room, Bedroom, etc.)
17. What information and/or knowledge clips (regarding diabetic foot problems) would you still like to see in the Foot Check App?
18. Do you plan to continue using the Foot Check App in the future? If yes, what information and/or knowledge clips do you miss? Why yes/no?
19. Are there any features you would like to see added to the Foot Check App that would improve your experience?
20. If you have ever been asked to take a picture of your feet with the Foot Check App: Did you find it easy to do?
21. If you have ever been asked to take a picture of your feet with the Foot Check App: How did you find taking a picture of your feet?
22. Can you briefly explain what was easy or difficult about taking a picture of your feet?
23. Do you get help using the Foot Check App? If so, from whom?
24. What information and/or knowledge clips (regarding diabetic foot problems) would you like to see in the Foot Check App?
25. At what time of day do you usually use the Foot Check App? (indicate what occurs most often)
26. Can you explain how the Foot Check App has made you more or less aware? This person (or persons) helps me with the use of the Voetencheck App
27. Would you recommend other people to use the Voetencheck App?
28. May we contact you for a telephone conversation about your experiences with the Voetencheck App?
