# Supplementary material for: Self-Monitoring Risk Factors for Diabetic Foot Ulceration With the Feetchecker App: Mixed Methods Study
Source: JMIR Form Res. 2026 May 27;10:e80769. doi: 10.2196/80769 (PMC13215667; doi:10.2196/80769)
Supplement: Multimedia Appendix 6 [file formative-v10-e80769-s006.docx]

Interview Guide - Participants

This is the document to collect questions for the semi-structured interviews with Participants. The interviews were in Dutch. Questions were often followed up with questions like; “what do you mean by that?”, “Could you elaborate this?”, etc.

**Introduction**

· Thank you for your participation.

· Explain the setup and duration of the interview

· Ask for consent for recording and indicate that it will be processed anonymously.

· Write down patient ID.

• What does your day look like? How active are you?

• Do you have someone around you who helps you with your health?

• Have you been visiting a podiatrist for a long time?

• Have you ever had a wound/problem on your feet?

• What did you think when your podiatrist suggested using the app?

• What motivates you to use the app?

• What did you think of using it? What did you like? What held you back?

• [data] – you've used it 7 times, can you talk us through it?

• Do you need help using the app? How does it work?

• Do you still use the app? If not, how often do you inspect your feet yourself?

o Why would you like to continue using it? Why not?

Interview Guide - Podotherapeuten

This is the document to collect questions for the semi-structured interviews with Podiatrists. Interviews were done in Dutch and translated.

**Introduction (5 min.)**

- Briefly tell us about the study and what we're working on.
- Request permission for the interview and audio recording.

**Demographics.**

- Can you briefly tell us about yourself? What do you do, and how long have you been doing it? Do you have any experience with diabetes care? Are you familiar with the Voetencheck app?

**General information about eHealth and digital care provision. (5 min.)**

- What kind of digital care support tools do you use in your daily practice?
- Can you describe your experiences with these tools? Can you describe some positive and/or negative aspects?
- What are your thoughts on the idea of ​​"remote care," or monitoring a patient's health remotely via digital means?
- How do you view people's "responsibility" for monitoring their own health? When does this work well? When doesn't it?

**Diabetes Feetchecker App (20 min.)**

- Do you have experience with the Foot Check App? Can you describe your experiences with it?
- Are you familiar with features such as the Knowledge Clips, timeline, and photo-taking functionality?
- What are the positive aspects of the app? What could be improved or would you like to see?
- What would be some quick improvements?
- Where do you see long-term potential?
- Do you discuss the Foot Check App during consultations?
- How do you approach this? What do you share? When don't you share it?
- Do you have patients who use the Foot Check App? Do they talk about it during consultations? What do they share?
- Do you see differences in suitability between patients in their experience with digital tools?
- In your experience, does a patient's social or cultural background affect their use of the Foot Check app?
- Do you have patients who made an appointment after submitting a photo via the Foot Check app? Do you discuss this during a consultation?
- Have you personally inspected Voetencheck photos in the electronic health record (EHR)?Do you have time for this?
- How do you approach this? Can you describe your workflow?
- Which aspects work well? Which don't work so well? What functions or aspects would you like to see in the EHR?
- What kind of data would you like to see about a patient? What information do you miss during a consultation/appointment?
- Do you see value in these types of systems? Where do you think it lies?
- How do you envision the use of digital healthcare tools like the Voetencheck app in the future?
