# Supplementary material for: Self-Monitoring Risk Factors for Diabetic Foot Ulceration With the Feetchecker App: Mixed Methods Study
Source: JMIR Form Res. 2026 May 27;10:e80769. doi: 10.2196/80769 (PMC13215667; doi:10.2196/80769)
Supplement: Multimedia Appendix 7 [file formative-v10-e80769-s007.docx]

**Table S1.** Demographics per participant.

| **P^1^** | **Age** | **Sex** | **ZP^2^** | **Which Check Question was answered with “YES”.^3^** |
| --- | --- | --- | --- | --- |
| P01 | 74 | m | 4 | - |
| P02 | 81 | m | 4 | - |
| P03 | 73 | m | 3 | - |
| P04 | 68 | f | 4 | - |
| P05 | 69 | m | 3 | (Q7) Do you have a wound of small wound on your foot? |
| P06 | 63 | m | - | - |
| P07 | 67 | f | 4 | - |
| P08 | 75 | m | - | - |
| P09 | 75 | m | 4 | - |
| P10 | 78 | m | 3 | - |
| P11 | 80 | m | 3 | - |
| P12 | 85 | m | 3 | - |
| P13 | 74 | f | 4 | - |
| P14 | 64 | m | 4 | - |
| P15 | 71 | m | 3 | - |
| P16 | 58 | m | 3 | - |
| P17 | 76 | m | 3 | (Q5) Do you see a blue discoloration underneath your nails? (2 times) |
| P18 | 80 | m | 3 | (Q2) Do you have red spots on your feet?  (Q3) Do you have red spots on your feet or toes that start or get worse when wearing your shoes? |
| P19 | 72 | m | 3 | (Q8) Is your foot red, thick and swollen? |
| P20 | 62 | m | 4 | (Q2) Do you have red spots on your feet?  (Q3) Do you have red spots on your feet or toes that start or get worse when wearing your shoes?  (Q9) Is one of your feet warmer or colder than the other foot? |
| P21 | 53 | m | 3 | - |
| P22 | 82 | m | 1 | - |
| P23 | - | - | - | - |
| P24 | 54 | m | 4 | (Q9) Is one of your feet warmer or colder than the other foot? |

Abbreviations:

1. P = patient number
2. ZP = Zorg Profiel / Care Profile. This is a Dutch risk classification by the podiatrist on the care package needed by the patient. ZP1 is low risk (Sims 1), ZP2 is a Sims 2 classification and the diagnosis of decreased sensation in the feet, reduced circulation. ZP3 is Sims 2 classification and the diagnosis of decreased sensation in the feet, reduced circulation *and* pressure sores. ZP4 is a Sims 3 classification and the presence of a previous foot wound or amputation, or this is a case of inactive Charcot foot or renal replacement therapy (dialysis).
3. Feetchecker **Q**uestions (See Appendix A1):
4. Do you have soft (white) skin between your toes?
5. Do you have red spots on your feet?
6. Do you have red spots on your feet or toes that begin or get worse while wearing your shoes?
7. Do you have calluses with a dark discoloration on your foot?
8. Do you see a blue discoloration underneath your toenails?
9. Do you see other discoloration (different from your skin color) on your foot?
10. Do you have a wound or small wound on your foot?
11. Is your foot red, thick and swollen?
12. Is on of your feet warmer or colder than the other foot?
